# Superabsorbent Polymers: Innovations in Ecology, Environmental, and Diverse Applications

**DOI:** 10.3390/ma18040823

**Published:** 2025-02-13

**Authors:** Qingya Niu, Jiayin Xie, Jiayan Li, Zaixu An, Huijie Xiao, Xiaoyuan Zhang, Zhiqiang Su, Zhichao Wang

**Affiliations:** 1 State Key Laboratory of Chemical Resource Engineering, Beijing Key Laboratory of Advanced Functional Polymer Composites, Beijing University of Chemical Technology, Beijing 100029, China; 2 School of Soil and Water Conservation, Beijing Forestry University, Beijing 100083, China; 3 Precision Forestry Key Laboratory of Beijing, Beijing Forestry University, Beijing 100083, China

**Keywords:** superabsorbent polymers, water absorbency, environmental sustainability, drought mitigation, arid environments

## Abstract

Significant progress has been achieved in the development of superabsorbent polymers (SAPs), focusing on enhancing their performance and expanding their applications. Efforts are particularly directed at increasing water absorbency while promoting environmental sustainability. Biodegradable materials such as starch and potassium humate have been successfully integrated with SAPs for desert greening, improving water retention, salt resistance, and seedling survival. The inclusion of nutrient-rich organic-inorganic composites further enhances the durability, efficiency, and recyclability of SAPs. In drought mitigation, polymeric absorbent resins such as polyacrylamide and starch-grafted acrylates have shown efficacy in ameliorating soil conditions and fostering plant growth. In arid environments, agents enriched with humic acid and bentonite contribute to improved soil aeration and water retention, creating optimal conditions for plant establishment. Additionally, the adoption of innovative waste management solutions has led to the production of amphiphilic SAPs from residual sludge, effectively addressing soil nutrient deficiencies and environmental pollution. In the food industry, SAPs containing protease, tea polyphenols, and chitosan exhibit potential for enhancing the stability and quality of seafood products. These advancements highlight the growing relevance of structural optimization approaches in SAP development across diverse applications and underline the importance of continued innovation in these fields. As novel materials emerge and environmental challenges intensify, the potential applications of SAPs are anticipated to expand significantly.

## 1. Introduction

Superabsorbent polymers (SAPs) hold significant potential for applications in ecological systems. In agriculture, SAPs can serve as water-retaining agents, absorbents, slow-release carriers, and seed coating materials, enhancing water efficiency and nutrient delivery [1]. In soil management, they function as soil conditioners, improving soil water retention, fertility, and biological activity, ultimately increasing plant survival rates [2]. Due to their high water absorption and retention capacities, SAPs are also effectively employed in desertification control and ecological restoration projects [3,4]. Their use reduces the reliance on artificial irrigation, mitigates water scarcity challenges, and minimizes adverse environmental impacts.

By addressing the issues of plant growth and survival in arid and drought-prone regions, SAPs contribute significantly to the advancement of desert reclamation and ecological restoration initiatives. The raw materials for SAPs can be broadly categorized into two types: natural superabsorbent polymers and synthetic polymers [5]. Natural superabsorbent polymers primarily include cellulose [6], chitosan, starch [7], proteins, alginates [8], and biomass. Synthetic polymers, on the other hand, mainly consist of polyacrylate, polyamino acids, and polyaspartate, among others.

The design methods for SAPs primarily include bulk polymerization [9], solution polymerization [10], inverse suspension polymerization [11], and radiation polymerization [12,13]. Bulk polymerization involves the use of monomers and initiators, offering simplicity in operation and eliminating the need for complex equipment. However, it faces challenges such as difficulties in heat dissipation and temperature control. To address these issues, solution polymerization can be employed, which provides better heat conductivity and precise temperature control. However, its low monomer concentration leads to a reduction in product yield. Inverse suspension polymerization effectively overcomes the limitations of the previous two methods and improves product quality. Radiation polymerization, meanwhile, demonstrates superior reaction efficiency and resource utilization compared to the other three methods.

Zhao et al.’s study indicates that at pH = 6, the PAA-SS-DE resin achieves maximum adsorption capacities for Cu^2+^ and Pb^2+^, reaching 236.0 mg/g and 247.2 mg/g, respectively. In contrast, the adsorption capacities of non-polymeric materials such as activated carbon typically do not exceed 100 mg/g. It can thus be inferred that the maximum adsorption capacity of SAP is significantly higher than that of other non-polymeric adsorbents [14]. Superabsorbent polymers (SAP) are functional macromolecular materials synthesized through polymerization reactions of unsaturated olefin monomers (such as acrylic acid, acrylamide, etc.) with the addition of crosslinking agents and initiators. Their preparation requires precise control of reaction conditions and entails relatively high economic costs. Non-polymeric materials like activated carbon have a wide range of raw material sources, often derived from wood chips, fruit shells, coal dust, etc., resulting in lower raw material costs and a more mature preparation process. Therefore, the economic cost of SAP is generally higher than that of other non-polymeric adsorbents.

SAPs, characterized by a three-dimensional network structure formed by water and hydrophilic polymer chains, exhibit excellent water retention and absorption properties due to their spatial configuration [15,16]. This makes them highly promising for ecological applications. Based on this, this review will discuss the following key aspects: First, it will introduce the definition and types of superabsorbent polymers. Next, it will explore their design methods, such as bulk polymerization, solution polymerization, inverse suspension polymerization, and radiation polymerization. Finally, the review will analyze the ecological applications of SAPs, including soil enhancement, agricultural improvement, and desertification control. Through these discussions, the review aims to highlight the significant value of superabsorbent polymers in sustainable development.

**Scheme 1:**
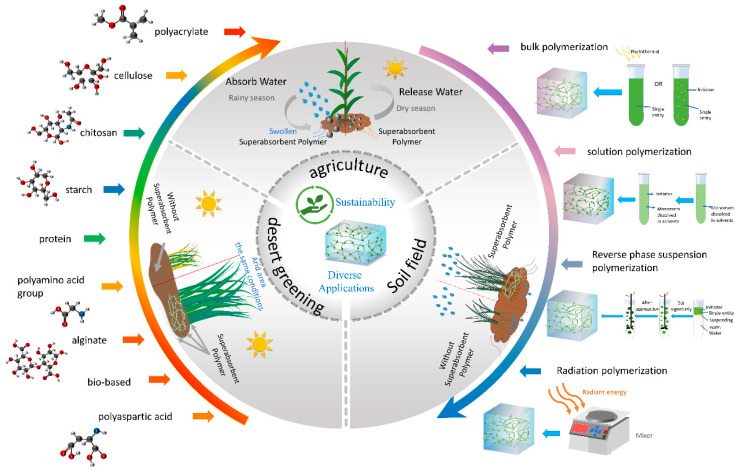
Classification, preparation, and applications of SAPs.

This review provides a more comprehensive and in-depth discussion on the classification and preparation of SAP and explores current cutting-edge research, such as the integration of nanotechnology with SAP (Scheme 1). In terms of application fields, the review focuses more on summarizing its applications in the ecological domain, addressing environmental issues from the perspective of sustainable development strategies. Firstly, the paper covers nine different types of SAP synthesized from various raw materials, elaborating in detail on both the definitions of these materials and the synthesis and performance characteristics of their corresponding SAPs. Previous reviews often concentrated solely on the performance of SAPs synthesized from a single raw material [17]. Among them, bio-based superabsorbent copolymers derived from renewable resources can serve as effective additives to improve the performance of cement-based materials, thereby reducing resource utilization. Secondly, this review conducts a more comprehensive and in-depth exploration of the preparation methods for SAP. The four preparation methods are analyzed by defining each preparation technique and discussing the advantages of using each technique to synthesize SAP [18]. This approach differs from previous reviews, which typically focused on one or two preparation methods. Further elaboration on the production processes aids in process optimization, thereby enhancing production efficiency and reducing energy consumption. Finally, the paper examines the application value of SAP in agriculture, desert greening, and soil improvement and proposes a “farmer-centered” ecological strategic concept aimed at maximizing agricultural net income while minimizing the ecological footprint [19].

## 2. Design of SAPs

### 2.1. Structural Characteristics of SAPs

SAPs are a class of functional polymers [17] known for their exceptional water absorption and retention properties [18]. These polymers possess a three-dimensional network structure, formed by crosslinked polymer networks composed of water-soluble monomer units. When placed in an aqueous solution, SAPs can significantly swell and absorb liquids up to several hundred times their dry weight [15,16]. This water absorption behavior is attributed to the osmotic pressure and the presence of abundant hydrophilic functional groups on the polymer molecular chains [19,20,21,22,23,24,25,26].

**Figure 1:**
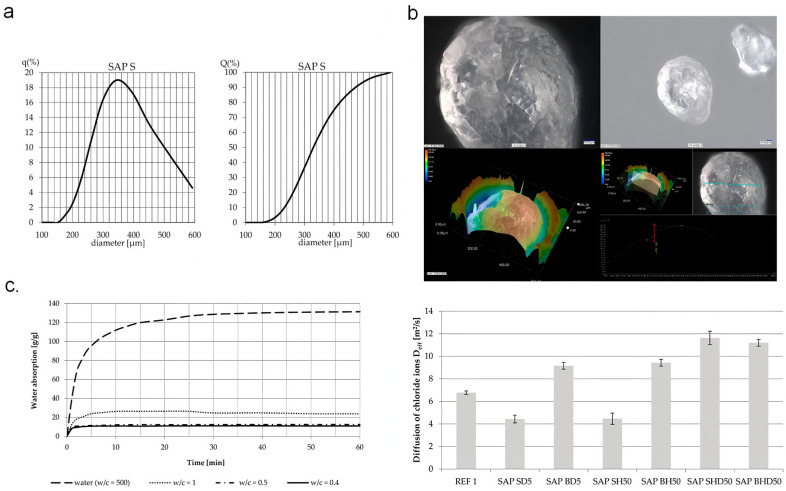
Effect of the water-to-cement ratio on chloride diffusion in SAP-modified concrete hydrogels: (**a**) Particle size distribution of SAP S measured by laser diffraction. (**b**) Unsaturated SAPS particles under drying conditions. (**c**) Variation of water absorption capacity over time under different water-to-cement ratios and chloride ion diffusion coefficients of concrete series. Reproduced with permission from ref. [31] © MDPI AG, CC BY.

SAPs are high molecular weight polymers formed by the grafting, copolymerization, and crosslinking of water-soluble monomers under specific conditions. The molecular structure of SAPs contains both hydrophobic and numerous hydrophilic groups [27]. Hydrophilic groups, such as hydroxyl, carboxyl, and amide groups, form a three-dimensional network structure within the polymer, providing SAPs with their water absorption and retention capabilities [28]. The structure of superabsorbent polymers is composed of a crosslinked hydrogel network [29]. Under specific water absorption conditions, SAPs can absorb water due to the osmotic pressure generated by the aggregation of ions within the polymer structure [30]. Water is absorbed into the polymer network of SAPs, causing an expansion in the particle volume, which facilitates the dispersion of ions within the structure (Figure 1). As the polymer network swells, the osmotic pressure gradually decreases [31].

SAPs exhibit voids and tubular structures, forming a network with a large specific surface area and extensive cross-linking, which contributes to their strong water absorption capabilities. The type and concentration of cross-linking agents influence the cross-linking density and the compactness of the internal structure of the super-absorbent polymer, thereby affecting the gel’s mechanical properties. At low cross-linking densities, SAPs fail to develop an effective spatial network, resulting in weak water absorption. Increasing the amount of cross-linking agent or the number of cross-linking points enhances the cross-linking density of SAPs, thereby moderately improving the polymer’s water absorption capacity. However, excessively high cross-linking densities can shorten the chain segments of ester bonds at the cross-linking points within the network, reducing the number of water molecules that SAPs can retain and thus decreasing water absorption. Therefore, an optimal degree of cross-linking exists that maximizes the water absorption of SAPs. In non-polar systems, such as organic solutions, SAPs demonstrate strong hydrophilicity; however, the absorption rate of organic solutions is generally low at low cross-linking densities. Furthermore, high cross-linking densities increase the restriction on organic molecules entering the network, leading to an even lower absorption rate.

### 2.2. Classification of SAPs

The water absorption performance of non-ionic SAPs is primarily attributed to the abundant hydrophilic functional groups on the polymer chains [32,33], which interact with water molecules through van der Waals forces, dipole interactions, or hydrogen bonding. Accordingly, SAPs can be classified into two main categories—non-ionic and ionic (including anionic and cationic)—based on whether their crosslinked network contains charged functional groups.

SAPs can also be classified according to the types of monomers that constitute their chemical structure. Most SAPs fall into one of the following categories: crosslinked polyacrylate and polyacrylamide, crosslinked copolymers of acrylate and acrylamide, and polyacrylate grafted onto natural polymers (such as cellulose, starch, chitosan, etc.) [34].

Given the wide variety of monomers and macromolecular structures, a range of SAP types can be synthesized. From the perspective of raw material sources, all SAPs can be classified into two major categories: fossil fuel-based and bio-based. Additionally, considering their biodegradability, SAPs are further subdivided into four main categories. This classification approach is useful for assessing the environmental impact and sustainability of SAPs [18].

SAPs are typically classified into two major categories based on their raw material sources: natural superabsorbent polymers and synthetic ones [5]. Synthetic superabsorbent polymers, particularly acrylate-based polymers, are the most widely applied in the market. These polymers are renowned for their excellent water absorption and retention capabilities; however, a limitation is their poor biodegradability [35]. Natural superabsorbent polymers primarily include chitosan and its derivatives-based SAPs [32,36], cellulose-based SAPs [37], starch-based SAPs [35], protein-based SAPs [38], amino acid-based SAPs [39], alginate-based SAPs [40], and bio-based SAPs.

Yang et al. investigated the crack sealing efficiency (r) of SAPs in NaCl solutions with varying concentrations and found that when the NaCl concentration is below 0.2 mol/L, the r value remains almost unchanged. However, when the NaCl concentration exceeds 0.2 mol/L, the r value decreases as the concentration increases. Therefore, controlling the NaCl concentration below 0.2 mol/L results in relatively higher crack sealing efficiency of SAPs [41]. With the increase in NaCl solution concentration, the presence of cations in the external solution reduces the osmotic pressure difference between the interior and exterior of the highly absorbent polymer network. This reduction prevents water from entering the polymer network, thereby continuously diminishing the water absorption capacity of SAP. When the ion concentration in the solution becomes sufficiently high, SAP may lose its water absorption capability.

#### 2.2.1. Polyacrylate-Based SAPs

Polyacrylate is a polymer synthesized from acrylate monomers [42,43], renowned for its excellent mechanical properties, anti-aging characteristics, light stability (non-yellowing), and good water resistance. As a highly important adhesive, polyacrylate is widely used in applications such as furniture coatings, automotive paint finishes, and adhesive products [44]. However, polyacrylate also has certain limitations, including insufficient resistance to organic solvents and certain chemicals, poor toughness and abrasion resistance, and potential issues of increased viscosity at high temperatures and brittleness at low temperatures [42].

Polyacrylate-based superabsorbent materials are highly favored in the market due to their remarkable water absorption performance (high absorption multiples and rapid absorption rates), readily available raw materials, simplified production processes, cost-effectiveness, and stable product quality [45]. Free radical polymerization is the primary method for synthesizing polyacrylate-based SAPs, with traditional synthesis techniques involving four different polymerization methods: bulk polymerization, solution polymerization, inverse emulsion polymerization, and inverse suspension polymerization [46]. Each of these methods offers distinct advantages, providing diverse approaches for various application scenarios [47].

#### 2.2.2. Cellulose-Based SAPs

**Figure 2:**
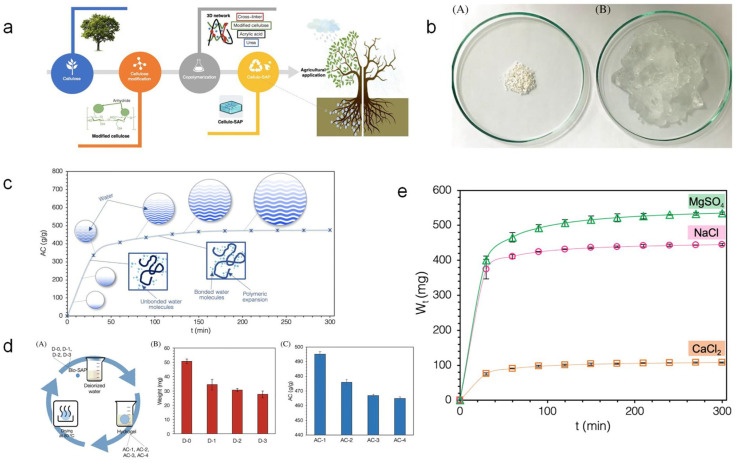
Synthesis and performance study of cellulose-based SAPs: (**a**) Production and applications of cellulose-based SAPs. (**b**) Conversion of cellulose-based superabsorbent polymers into hydrogels through physical methods. (**A**) After reaching absorption capacity (AC) equilibrium in deionized water, completely dry 0.5 g cellulose-SAP samples and (**B**) hydrogel. (**c**) Changes in Swelling Behavior and Absorption Capacity (AC) of Cellulose Superabsorbent Polymers in Deionized Water over Time (t). (**d**) Effect of absorption-desorption on weight and absorptive capacity (AC): (**A**) method scheme, (**B**) weight of the entire expansion-drying cycle, and (**C**) AC of the entire expansion-drying cycle. (**e**) Absorbed liquid weight (Wt) by immersion in 1.0 mol/L MgSO_4_, NaCl, and CaCl solutions of cellulose superabsorbent polymers and time (t). Thermogravimetric analysis of cellulose-based SAPs. Reproduced with permission from ref. [48] © 2022 Canadian Society for Chemical Engineering.

Cellulose, as a natural polymer compound, is a major component of plant cell walls and is widely distributed in nature. It is a renewable and abundant resource [6]. Photosynthesis is the primary method for cellulose synthesis (Figure 2), and it is estimated that plants produce approximately 1.5 × 10^11^ tons of cellulose annually [48]. Cellulose is a linear polysaccharide macromolecule composed of D-glucopyranose units connected by β-1,4-glycosidic bonds, with a molecular formula of (C_6_H_10_O_5_)n, where n represents the degree of polymerization [6]. In addition to its widespread distribution in nature, cellulose possesses the following characteristics: first, its production and supply are seasonal; second, the production scale of cellulose can be flexibly adjusted according to demand; third, cellulose exists in various forms, with differences in composition and structure of fibers, hemicellulose, and lignin depending on the source; finally, cellulose is relatively inexpensive and often considered a waste product. These characteristics make cellulose a promising material with extensive potential applications across various fields [49].

Cellulose-based SAPs are prepared via a free radical polymerization reaction [50], where pre-modified Sigmacell-type cellulose (20 type, 20 microns) is co-polymerized with urea and acrylic acid (Sigma-Aldrich) in a mixed solution, under the influence of initiators and crosslinking agents [51]. Cellulose-based superabsorbent resins feature rigid and flat molecular chains with abundant hydroxyl groups [52]. Similar to starch, cellulose can be derivatized to achieve a higher degree of substitution, reducing the number of washing steps and post-drying treatments. Compared to synthetic superabsorbent resins, cellulose-based SAPs have poorer water absorption capacity but better salt resistance [53]. These cellulose-based superabsorbent resins not only meet the higher performance requirements in agriculture and forestry, such as salt resistance, gel strength, and biodegradability [54], but also have broad application potential in scenarios where water absorption requirements are relatively flexible [55]. Therefore, the synthesis of cellulose-based superabsorbent resins represents an important direction for sustainable and green progress in the field of superabsorbent polymers [52]. When cellulose-based SAP is at 40–60% humidity, the degradation rate within 90 days is 30–50%, and if the humidity is not suitable, the degradation rate decreases; when cellulose-based SAP is at 20–30 °C, the degradation rate is 40–60% in 90 days, and the temperature is too high or too low to change the degradation rate; in soils rich in cellulose-decomposing bacteria, the degradation rate of cellulose-based SAP is 70–90% in 120 days, but only 30–50% when lacking.

#### 2.2.3. Chitosan-Based SAPs

Chitosan is prepared through the deacetylation reaction of chitin and is one of the most abundant natural biopolymers on Earth [56]. Chitin is widely distributed in nature and is a renewable and highly abundant natural polymer [57]. Due to its unique molecular structure and significant biological activity, chitosan and its derivatives are considered to be highly promising biomaterials [56]. Chemically modified chitosan has shown clear advantages in improving solubility, enhancing biocompatibility, promoting biodegradation, maintaining stability, and increasing the reactivity of functional groups [58]. These advantageous properties have greatly expanded the research and application scope of chitosan in drug delivery systems and biomedical engineering [59].

Chitosan is rich in highly active amino and hydroxyl groups [60], which enables it to undergo chemical modification under relatively mild conditions, further leading to the synthesis of chitosan-based superabsorbent resins [61]. Through the principle of free radical polymerization, water-soluble chitosan derivatives have been successfully synthesized into SAPs, which not only exhibit excellent water retention and absorption properties but also find extensive applications in ecological fields [62]. In 50–70% humidity, the degradation rate of chitosan-based SAP is about 40–60% for 60–90 days, and the degradation rate decreases when the humidity is abnormal; at 20–30 °C, the degradation rate of chitosan-based SAP is 30–50% for 60 days, and the temperature abnormality affects the degradation rate of enzyme activity. In soils with a large number of chitosan-active microorganisms, the degradation rate of chitosan-based SAP is 60–80% for 75 days, while the microorganisms are less than 30–40%.

#### 2.2.4. Starch-Based SAPs

Starch is one of the carbohydrates synthesized by higher plants and plays a crucial role within the plant body [7]. In addition to promoting plant growth, development, and reproduction, starch is the most abundant biopolymer in nature after cellulose. As a major component of food and feed, starch is also an important raw material for bioethanol and other bioenergy products [63]. Starch consists of two polysaccharides, amylose and amylopectin, which exist in plant cells in particles of varying shapes and sizes. These particles feature a concentric ring structure alternating between semi-crystalline and amorphous phases, with the characteristic “Maltese cross” pattern observable under polarized light microscopy [64].

Due to the superior degradation performance and environmental compatibility of starch-based superabsorbent resins, it has become a research focus in the field of superabsorbent polymers [37]. However, these resins have relatively weak resistance to degradation, and their reusability significantly decreases, which greatly limits their application in key areas [37]. Currently, free radical-induced polymerization is the primary method for synthesizing starch-based superabsorbent polymers by grafting hydrophilic monomers (such as acrylic acid and acrylamide(Sigma Aldrich Chemicals) onto starch molecules. This method can be classified into two types: one involves grafting co-polymerization of starch with a single monomer, while the other achieves multi-monomer grafting co-polymerization with starch [65]. Based on the principle of free radical graft polymerization, SAP can be prepared by grafting partially neutralized acrylic acid onto starch. The CST-PRP-SAP samples studied by Bai et al. exhibited excellent water retention and phosphorus release properties, with high water absorption rates. As the PRP content increased and the neutralization degree decreased, both the cumulative phosphorus release amount and release rate of the CST-PRP-SAP samples increased [66]. Soil moisture retention changes with and hydrogel conditioner under different watering intervals [67,68,69,70,71,72] The degradation rate of starch-based SAP is 30–50% within 30–60 days under 60–80% humidity, and the degradation rate decreases when the humidity deviates from this range; 25–35 °C is conducive to the degradation of starch-based SAP, and the degradation rate is 40–60% within 30 days, and the degradation rate decreases greatly when the temperature is too high or too low; the degradation rate of starch-based SAP in soil rich in amylase-producing bacteria is 50–70% within 45 days, while only 20–30% when microorganisms are deficient.

#### 2.2.5. Protein-Based SAPs

Proteins are essential substances for maintaining life activities, with amino acids being their basic building units. The biological functions of proteins are mainly influenced by their three-dimensional spatial structure [73]. Typically, proteins with similar structures exhibit comparable biological activities, and proteins performing similar functions often share structural similarities [74]. Based on their functional characteristics, proteins can be broadly classified into several categories, including enzymes, transport proteins, storage proteins, contractile proteins, toxin proteins, antibodies, hormone proteins, and structural proteins. These proteins play diverse biological roles within organisms, such as catalyzing metabolic reactions, exerting hormonal effects, transporting molecules, recognizing signals, and participating in the regulation and control of biological processes. Different types of proteins work together to ensure the proper physiological functions and homeostasis of an organism.

Cuadri et al. investigated the macroscopic properties of SAPs based on soy protein isolate (SPI) and the correlation between these properties and the level of chemical modification of the protein. They found that, compared to the reference sample, acylated plastics demonstrated superior extensibility due to their higher water absorption capacity. The results indicated that SAPs prepared by chemically modifying SPI not only enhanced water absorption but also improved mechanical extensibility, providing important experimental evidence for the development of bio-based SAPs with good water absorption and mechanical properties [75].

#### 2.2.6. Poly(aminoacid)-Based SAPs

Polyamino acid hydrogels exhibit a unique secondary structural feature, which plays a crucial role in shaping their gel characteristics. To precisely regulate these gel properties, the composition of the polyamino acid’s constituent units, the length of the molecular chain, as well as the types and quantities of side chains or terminal groups, can be effectively controlled, thereby achieving the optimization of the secondary structure of polyamino acids and the customized regulation of gel properties [76].

In the process of constructing a hydrogel precursor (PMHU) with excellent self-healing and shear-thinning properties, poly-1-glutamic acid (PLGA) was employed as the core biodegradable matrix material. To enhance the functionality of this matrix, branched α-hydroxy-ω groups were cleverly introduced, and UPy units were integrated into the system through the bridge molecule -amino poly(ethylene glycol) (HAPEO). Moreover, to impart reactivity to the hydrogel precursor, ethyl acrylate poly(ethylene glycol) (MAPEG) was utilized as a source of double bonds, successfully incorporating double bonds into the structure. Through this carefully designed synthetic strategy, PMHU was successfully prepared. This material not only maintains good biodegradability but also demonstrates excellent self-healing ability and shear-thinning characteristics, providing new possibilities for smart materials and biomedical applications [77].

Zhao et al. used glutamic acid fermentation broth instead of pure glutamic acid powder for fermentation and studied the effects of centrifugation, ultraviolet treatment, and high-temperature treatment on the fermentation broth. They found that after centrifugation, the yield of γ-PGA decreased by 5%, but this did not affect the formation of the hydrogel. The results suggest that this method not only simplifies the separation process of γ-PGA fermentation broth and reduces production costs but also improves the performance of superabsorbent resins [78].

#### 2.2.7. Alginate-Based SAPs

Alginate is a polysaccharide derived from brown algae, known for its biodegradable, non-toxic, cost-effective, and gel-forming properties, as well as its antibacterial and anticancer activities. It is widely used in soil improvement, the food industry, and biomedicine [8,79]. In soil improvement, alginates and their derivatives have been proven to enhance soil water retention, increase nutrient utilization efficiency, optimize soil structure, and serve as effective ground cover materials [79]. In the food industry, due to their antibacterial, anticancer, and probiotic properties, alginates improve the texture and taste of food while also extending the shelf life of food products [80]. In the pharmaceutical industry, the gelation properties and absorbency of alginates make them ideal materials for wound treatment [8].

Alginate itself has good biocompatibility, low toxicity, low cost, and wide applicability. Materials made from alginate-based compounds exhibit excellent water absorption properties [45]. Sodium alginate-based SAP shows good agricultural benefits under drought conditions, capable of retaining moisture and nutrients. These materials cause minimal environmental pollution and are highly beneficial for agricultural development, with promising prospects [2].

**Figure 3:**
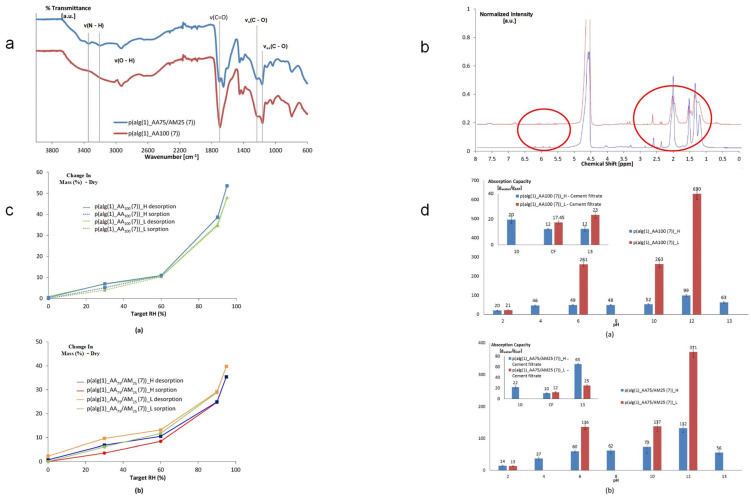
Performance characterization of SAP based on methacrylated alginate and acrylic acid monomers: (**a**). Characterization of SAP by attenuated total reflectance infrared (ATR-IR) spectroscopy. (**b**). High-resolution magic angle spinning (MAS) spectroscopy of SAP.The red circle indicates that after polymerization, the peak shifts to a lower ppm range (1.5–2.5 ppm). (**c**). Hygroscopicity was determined through dynamic vapor sorption measurements. (**a**) Moisture Absorption of p(alg(1)_AA100(7))_H and p(alg(1)_AA100(7))_L by Dynamic Vapor Adsorption Measurement (**b**) Moisture absorption of p(alg(1)_AA75/AM25(7)))_H and p(alg(1)_AA75/AM25(7))_L by dynamic vapor adsorption measurement (**d**). Comparison of swelling abilities of SAP in aqueous solutions at different pH values (acidic and alkaline) and cement filtrate solutions. (**a**) Comparison of the swelling capacity of p(alg(1)_AA100(7))_H and p(alg(1)_AA100(7))_L in aqueous solutions at different pH values and (acidified and alkaline) cement filtrate solutions (illustration). (**b**) Comparison of the swelling capacity of p(alg(1)_AA75/AM25(7))_H and p(alg(1)_AA75/AM25(7))_L in aqueous solutions and (acidified/alkaline) cement filtrate solutions (illustration). Reproduced with permission from ref. [81] © 2017 Elsevier Ltd.

Arn Mignon et al. studied the applications of polysaccharide-based (especially alginate-based) SAPs in drug delivery and self-healing concrete (Figure 3). They discovered the effectiveness of calcium alginate and methacrylated alginate combined with acrylic acid. The results showed that by altering the degree of methacrylation and using a combination of acrylic acid and acrylamide, the properties of the SAP could be significantly affected. The material exhibited a high gel fraction and an excellent swelling ability of up to 630 g water/g SAP, particularly for superabsorbent polymers with low substitution degrees. Furthermore, the SAP showed limited hydrolysis in aqueous and cement filtrate solutions [81].

#### 2.2.8. Biopolymer-Based SAPs

**Figure 4:**
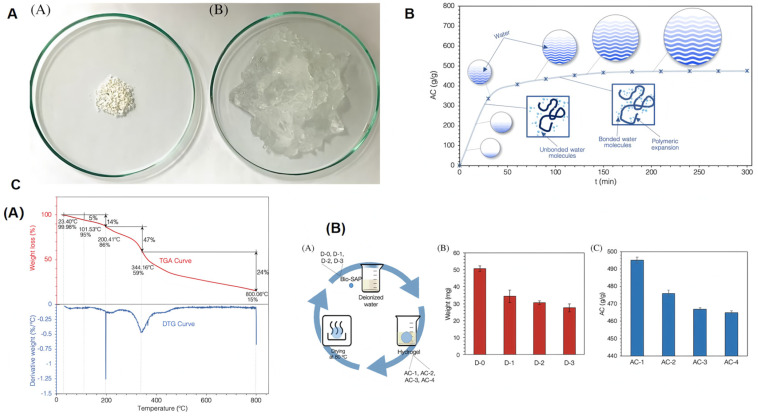
Study on the synthesis and performance of cellulose-based SAPs: (**A**) Conversion of cellulose-based superabsorbent polymers into hydrogels via physical methods. (**B**) Swelling behavior and absorption capacity of cellulose-based SAPs in deionized water over time (t). (**C**) (**A**) Thermogravimetric analysis of cellulose-based SAPs. (**B**) Evaluation of the effect of repeated swelling cycles (**A**) of the dried sample in deionized water on the weight (**B**) and absorption capacity (**C**) of the cellulose-based superabsorbent polymer. Reproduced with permission from ref. [48] © 2022 Canadian Society for Chemical Engineering.

Biobased refers to a class of new materials manufactured using renewable biomass, including crops other than food, trees, other plants, and their residues and contents, as raw materials, using biological, chemical, or physical methods [82]. Biobased SAPs can be generated from natural polymers such as polysaccharides derived from various renewable resources. These materials have advantages such as being renewable, non-toxic, and rapidly biodegradable. Studies have shown that SAPs made from cellulose and other raw materials exhibit excellent water solubility, biocompatibility, and biodegradability [52]. Due to their significant advantages of high biodegradability and low toxicity (Figure 4), they are widely used in agriculture and landscaping, making them a green, environmentally friendly material with promising development prospects [48].

Biomass materials are widely sourced, cost-effective, environmentally friendly, easily degradable, and readily prepared, making them suitable for various applications. Research indicates that biomass hydrogels possess excellent biocompatibility and low toxicity [83], and that bio-based superabsorbents exhibit outstanding water absorption capacity. These materials can be used as active agent carriers and soil conditioners, making them an interesting polymer material [84].

Anastasia N. Aday and colleagues studied the synthesis and characterization of bio-based superabsorbent copolymers from κ-carrageenan and polyacrylic acid. They found that these bio-based SAPs exhibited an absorption rate of up to 438 g/g after 24 h in aqueous solutions and 94 g/g in ionic solutions. The results suggest that these bio-based superabsorbent copolymers could become effective additives for improving the performance of cement-based materials [85].

#### 2.2.9. Polycondensate-Based SAPs

Polyaspartic acid, a type of polyamino acid, is commonly referred to as PASP [86]. It is often found in mollusks and exhibits excellent water absorption properties due to its high content of carboxyl groups and unique spatial structure [17]. The biodegradability of PASP polymers is closely related to the types of enzymes and microorganisms present in the soil, and degradation under special conditions is also influenced by other environmental factors in the soil [86]. PASP-based superabsorbent resins, with their excellent biocompatibility, flexibility, and similarity to natural tissues, show great potential in various applications. They can be widely used as soil conditioners, hygiene products, and more. By using chemical crosslinkers and cyclic freeze-thaw techniques, a new type of PASP-based superabsorbent resin can be developed, which effectively improves its swelling behavior, reduces the toxicity of chemical crosslinkers, and promotes green development [87].

PASP-based SAPs are widely applied in the biomedical field due to their excellent biocompatibility. They can be used in tissue engineering scaffolds, as genetic material carriers, or for controlled drug release. Additionally, they are also used as sand stabilizers, water retention agents, and plant fertilizers [88].

## 3. Fabrication Technology of SAP

### 3.1. Bulk Polymerization

Bulk polymerization is a chemical reaction process where monomers (or low molecular weight precursors) undergo self-polymerization in the absence of solvents and external dispersing agents, triggered by initiators, light, heat, or radiation. In this process, the monomers directly form high molecular weight polymers through intermolecular bonding. Sometimes, auxiliary components such as colorants, plasticizers, and molecular weight regulators are added to adjust the properties of the polymer or impart specific attributes [89]. Bulk polymerization involves only the initiators and monomers, which are crucial for controlling the purity of copolymers. It is a versatile method that can be performed with a wide range of monomers and across a broad temperature range [90]. This characteristic makes bulk polymerization valuable in various fields such as materials science, polymer chemistry, and industrial production. Under specific conditions, by precisely controlling reaction parameters such as temperature, pressure, and the type and amount of initiators, fine-tuning of polymer molecular weight, structure, and performance can be achieved to meet diverse application needs.

Bulk polymerization is conducted in the absence of any solvents or dispersants, making it the simplest in terms of formulation. It is employed in the synthesis of most step-growth polymers and many types of chain-growth polymers. In the case of chain-growth reactions, the heat released can cause the reaction to become excessively vigorous and difficult to control unless effective cooling is implemented. Because the resulting polymers are pure, large castings can be prepared directly, and the molecular weight distribution can be easily modified by using chain transfer agents, bulk polymerization is the preferred method for the synthesis of both step-growth and chain-growth polymers [91].

A significant advantage of bulk polymerization lies in its simplicity, as it does not require complex, sophisticated equipment to efficiently synthesize high-purity polymer materials, especially in the case of polymers, making it a fundamental and straightforward method in the preparation of superabsorbent resins [92]. However, in some experimental studies, achieving polymer blocks with irregular shapes and sizes requires grinding and processing, which can be time-consuming and may lead to waste. Additionally, the lack of process control during polymerization may result in heterogeneous regions within the polymer matrix with low adsorption affinity for target molecules [93].

**Figure 5:**
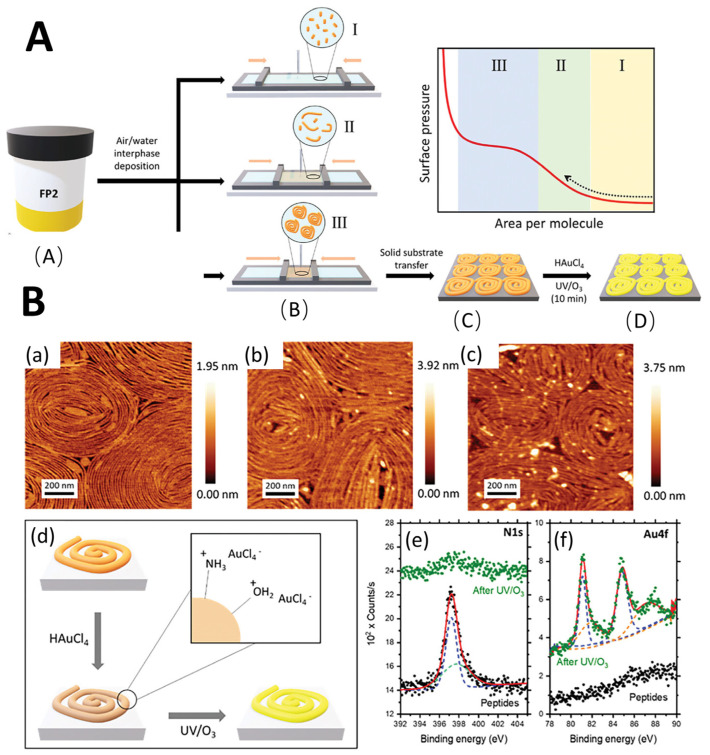
(**A**). Schematic summary of the experimental methods. (**A**) Peptide solutions were prepared in DMF. (**B**) deposited in air-water cross-linked enzymes and compressed using the LB setup. (**I**, **II**, **III**). The Langmuir trough barriers then exerted lateral, unidirectional compression at a constant speed until reaching surface pressures of 10, 20 or 30 mN·m^−1^. (**C**) transferred to the MICA matrix. (**D**) selectively impregnated with metal salts and removed with UV/O^3^ peptides. (**B**). Study on FP2 peptide: (**a**) AFM micrograph of pristine FP2 peptide placed on a mica substrate at 20 MN·m^−1^ surface pressure, (**b**) after immersion in Haucl4 solution for 30 min, and (**c**) after subsequent UV/O^3^ degradation treatment. (**d**) The interaction between the peptide fiber and the AU precursor salt in aqueous solution is schematically shown. (**e**) High-resolution XPS (black dots) of the original FP2 peptide of N 1s and (**f**) AU 4F and after inorganic incorporation and UV/O^3^ degradation (green dots). Reproduced with permission from ref. [96] © 2024 Wiley-VCH GmbH.

Qi Zhenming et al. investigated the construction of a flexible polymer network for potassium ion-based SAPs using potassium salt-based nanoparticles as physical crosslinking agents (Figure 5). They found that this novel K-SAP exhibited good water absorption and retention properties, as well as enhanced recyclability. This result suggests that the potassium ion-based SAPs obtained using this method have broad application prospects in agriculture and soil-related fields [38,94].

Qiuke Li et al. designed a pH-triggered self-assembling β-hairpin peptide SAP with dual biological functions of antibacterial activity and capture capability. They found that this nanopeptide exhibits antibacterial properties under acidic conditions by disrupting the integrity of bacterial membranes. At high concentrations, SAP induces bacterial aggregation accompanied by bacterial death, demonstrating good biocompatibility and therapeutic efficacy both in vivo and in vitro. This provides a new strategy for treating intestinal pathogen infections [95].

### 3.2. Solution Polymerization

The mechanism of solution polymerization involves processes initiated by ultraviolet irradiation or redox initiators, and during the solution polymerization process or crosslinking reactions, the crosslinking agent and the copolymer of two natural monomers are mixed [82].

Compared to melt condensation methods, the products produced by solution polymerization have higher molecular weights and better solubility in organic solvents [97]. During the solution polymerization process, when the final conversion rate is low, monomers with stronger conjugation are more likely to polymerize compared to others [98]. Therefore, in the practical application of solution polymerization, it is essential to consider both its advantages and limitations, adopting appropriate strategies to optimize process conditions and improve product quality and production efficiency.

Peng Wang et al. studied and characterized the materials’ structure and morphology using FTIR, SEM, and TGA. Under optimal synthesis techniques, they found that the superabsorbent polymers synthesized by solution polymerization exhibited excellent water retention and re-swelling capabilities. The results indicate that these outstanding properties make the successfully synthesized SAPs have broad application prospects in the agricultural field [99].

### 3.3. Inverse Suspension Polymerization

Inverse suspension polymerization is a polymerization method that uses oil as the primary dispersing medium [100]. In this process, water-soluble monomers, with the assistance of a suspending agent and through high-intensity stirring, can be effectively dispersed into fine aqueous droplets suspended in the oil phase. The initiating aqueous solution of monomers is introduced into a long vertical reactor, where it fills the monomer droplets within the continuous oil phase, thereby initiating the polymerization reaction as the droplets descend [101]. In such a specific microenvironment, inverse suspension polymerization efficiently controls the interactions and dispersion states between components, giving this method a unique advantage in the preparation of high-performance polymer materials.

Inverse suspension polymerization offers a stable polymerization process, effectively avoiding the phenomenon of product agglomeration into blocks and gel formation, thus simplifying subsequent processing steps. Additionally, this method promotes rapid dissipation of the reaction heat [11]. Inverse suspension polymerization is considered the polymerization of dispersed water-soluble monomers in a continuous organic matrix. Compared to other preparation methods, it yields fine powder-like products and allows for the adjustment of reaction conditions to easily control the particle size of the product [102]. This characteristic directly eliminates the need for grinding the granulated product, further improving production efficiency and product purity, and meeting the high standards required for high-quality polymer materials in both academic research and industrial applications.

**Figure 6:**
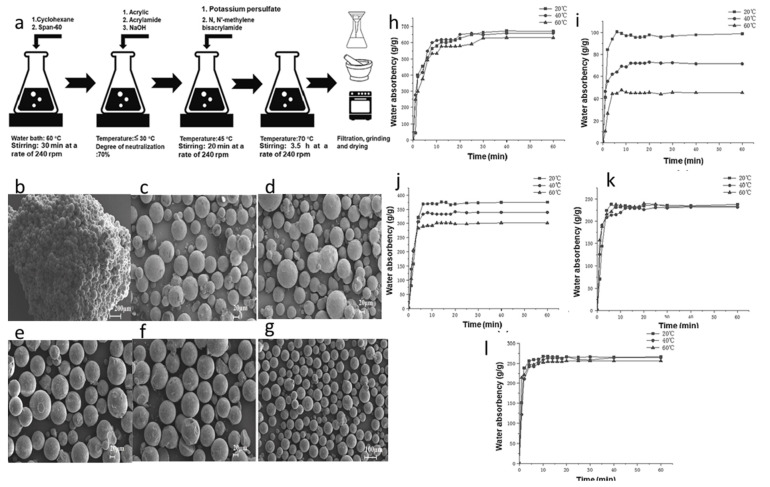
Preparation strategy and structural characterization of SAP synthesized based on Kaolin: (**a**) The synthesis process of kaolin-reinforced SAP. (**b**) The microstructure of SAP agglomerates before being crushed. (**c**–**g**) SAP with certain mechanical strength retains its spherical shape even after withstanding mechanical crushing pressure. (**h**,**i**) Water absorption rate of the synthesized SAP at different water temperatures. Reproduced with permission from ref. (**j**–**l**) The absorption of SAP with synthetic kaolin contents of 3.8%, 7.5% and 7.5% at different water temperatures. [101] © 2022 Elsevier Ltd.

Junying Lai et al. used 7.5% low-grade kaolin to synthesize SAP via inverse suspension polymerization (Figure 6). They found that the SAP synthesized by this method had a spherical shape with a uniform particle size distribution. After being saturated in deionized water, the SAP exhibited superior dispersibility, and its absorption was highly sensitive to both the temperature of the water and the relative humidity of the environment. The results indicated that low-grade kaolin enhanced the absorbency of the SAP [101].

### 3.4. Radiation Polymerization

Radiation polymerization technology is an efficient and environmentally friendly method for synthesizing hydrogels that can be directly triggered by radiation energy [12,13]. It effectively avoids the time-consuming issues associated with traditional chemical grafting methods, significantly improving reaction efficiency [103]. This advantage not only promotes the efficient use of resources but also reduces environmental burden, laying a solid foundation for the widespread application of radiation polymerization in both academic research and industrial production. Furthermore, compared to certain chemical initiators, radiation-induced polymerization offers several advantages, such as lower reaction temperatures, higher polymer purity, easier control over the reaction rate, a safer process, and the ability to interrupt the irradiation process [103].

Abd El-Mohdy et al. studied the synthesis of starch/(EG-co-MAA) hydrogels via radiation polymerization using ethylene glycol (Merck, Darmstadt, Germany) and grafted starch vinyl esters (El-Nasr Co. for Chemical Industries, Nasr City Cairo, Egypt). They found that the gel content of these hydrogels was influenced by the starch content, ethylene glycol content, the composition of vinyl esters, irradiation dose, and crosslinking density. The results indicated that this new superabsorbent polymer could be used for various applications [103].

### 3.5. Others

Lee et al. studied the preparation and characterization of surface-crosslinked spherical SAP particles. They found that by using a combination of inverse suspension polymerization and surface crosslinking with polycations such as PAMAM (Sigma-Aldrich, Milwaukee, WI, USA) and bPEI (Sigma-Aldrich, Milwaukee, WI, USA), the spherical SAP particles prepared by this method exhibited faster crosslinking at lower temperatures compared to traditional surface crosslinking methods. Additionally, the surface-crosslinked SAPs prepared using this method showed a higher swelling ratio and better performance. The results indicated that SAPs prepared using this method exhibited excellent performance and high efficiency, while surface crosslinking could prevent excessive swelling, providing a greater void volume fraction in gel beds filled with surface-crosslinked SAP particles and effectively reducing gel blockage effects [104].

## 4. Ecological Applications

### 4.1. Agriculture

SAP can be applied in agriculture in areas such as water-saving agriculture, horticultural water retention, and soil improvement. In agriculture, SAP can function as a water-retaining agent and soil conditioner, effectively improving soil fertility, promoting water absorption in the soil, and enhancing water use efficiency. It can also control the release of fertilizers, pesticides, etc., promote plant root absorption, reduce environmental pollution, and ensure good plant growth [45]. SAP can also be used as a seed additive, seed coating, root soaking, and stabilizer for plant growth regulators or controlled release protectants, which promote seedling growth, improve survival rates, and increase crop yields [1].

The most prominent feature of SAP is its high water absorption capacity. It is formed by polymerizing soluble monomers that contain a large number of hydrophilic groups, such as carboxyl and hydroxyl groups, with a three-dimensional network structure inside. SAP can absorb many times its own weight in water, giving it strong water-absorbing ability [105]. When SAP is incorporated into soil, it can absorb and retain water from irrigation or rainfall and gradually release it to plant roots, helping to conserve water and address water scarcity in arid regions [52]. This promotes crop growth.

Additionally, SAP exhibits excellent mechanical properties, including compressive strength, tensile strength, wear resistance, and superior elasticity. Experimental studies by Zhang et al. found that hydrogels containing organically modified montmorillonite (OMMT) demonstrate outstanding elasticity and extensibility. This is because the inorganic rigidity effectively enhances the hydrogel’s strength and improves the mechanical properties of the composite network, resulting in hydrogels with excellent mechanical performance [106]. In practical applications, when SAP is combined with water, it can store a large amount of moisture. Due to its superior mechanical properties, such as compressive strength, SAP can retain moisture even under high pressure and other special conditions, ensuring a moist soil environment. Additionally, it can resist compression and friction from soil particles, providing a certain degree of wear resistance. This helps maintain environmental moisture, optimizes its water absorption and retention capabilities, and promotes crop growth.

SAP has great application prospects in agriculture as an effective water-retaining agent. However, during its preparation, it is essential to fully consider its effectiveness in the soil, including factors such as salt and alkali tolerance, biodegradability, reusability, potential harm to the soil environment and crops, and cost-effectiveness [17]. The synthesis and application of SAPs should align with green and environmentally friendly principles to minimize environmental pollution. Their biodegradability should be fully leveraged by using natural polymer materials (such as starch, cellulose, etc.) as the backbone of SAPs, followed by the grafting of hydrophilic monomers to synthesize SAPs that are conducive to biodegradation. Similarly, synthetic polymers can be modified to achieve this synthesis [45]. This approach can partially reduce the generation of microplastics, which are carriers of pollution. In agricultural fields, microplastics (MP) may lead to changes in microbial communities and plant growth, with sources being quite widespread [107]. While the presence of MPs has certain positive effects on plant Cd uptake and soil Cd bioavailability, various non-degradable and biodegradable MPs can accumulate heavy metals, alter soil characteristics [108], and affect soil vitality, thereby impacting crop growth. Therefore, in agricultural production, the impact of MPs should be continuously monitored, biodegradable materials should be used more frequently, the sources of MPs should be reduced at their origins, environmental pollution should be minimized, and plant growth should be promoted.

**Figure 7:**
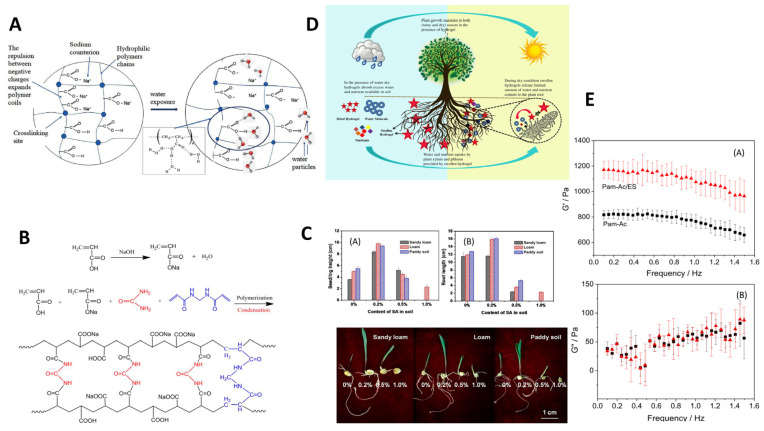
Crosslinking mechanism and high-performance characterization of superabsorbent polymers synthesized using modern nanotechnology: (**A**) General crosslinking mechanism between nanoparticles and polymer chains [38]. (**B**) Swelling kinetics of the synthesized hydrogel in distilled water and saline solution. (**C**) Swelling height and root length changed with content of SA in soil. (**D**) Schematical drawing of water and nutrient uptake. (**E**) Storage modulus (G′) (**A**) and loss modulus (G″) (**B**) of the synthesized hydrogel. Reproduced with permission from ref. [110] ©2017 Sociedade Brasileira de Química.

The efficient application of SAP in agriculture is beneficial for water retention and promoting plant growth [109]. By adding SAP, the flow rate of soil can be hindered, thus reducing permeability. Research indicates that using high water absorption hydrogels derived from agricultural waste as soil conditioners can promote agricultural production, creating a “farmer-centered” circular process that maximizes agricultural net income, reduces ecological footprints, minimizes environmental impacts, and promotes sustainable agricultural development (Figure 7) [110].

El Idrissi et al. studied the agricultural applications of polysaccharide-based SAPs and found that using SAP as a carrier to transport fertilizers to the soil results in a higher utilization rate compared to using fertilizers alone. This method can fully release fertility, reduce losses, minimize environmental pollution, effectively promote plant growth, and increase yields. The results show that SAP can serve as a carrier for agricultural chemicals, improve soil fertility, retain moisture, reduce environmental pollution, meet the needs of arid areas, and promote crop growth, making it widely applicable in the agricultural field [111].

### 4.2. Desert Greening

Due to its excellent water absorption and retention properties, SAP plays an important role in desert greening and reclamation. In arid regions, SAP can be applied to sandy soils to improve their water-holding capacity, thereby promoting plant growth and survival [1]. At the same time, SAP improves soil conditions, increases plant water absorption and utilization, and helps plants better adapt to dry environments [3]. It can be effectively used in desert greening projects to solve water scarcity problems in arid regions.

The excellent water absorption capacity of superabsorbent resins is closely related to their irregular three-dimensional network structure, which allows them to absorb water thousands of times their own weight. After absorbing water, they form hydrogels [54]. Due to their ability to provide and release moisture, they can improve plant survival rates under water-scarce conditions, making them ideal water-absorbing materials for desert greening [112].

SAPs can be widely used in desert reclamation. When mixed with sandy soil, they effectively improve the water retention ability of the soil and reduce water evaporation. However, the particle size and quantity of SAPs also affect the performance of the sandy soil. Therefore, the appropriate particle size of SAP should be selected, and its quantity controlled to improve performance and achieve successful desert greening [4].

**Figure 8:**
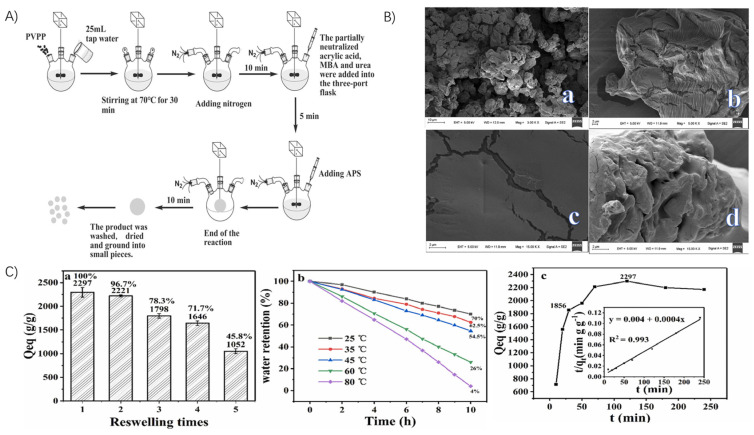
Preparation strategy and performance of PVPP-based superabsorbent polymer gels: (**A**) Flowchart of SAP preparation process. (**B**) (**a**) SEM image of PVPP surface; (**b**) SEM image of SAP surface before water absorption; (**c**) Local enlarged SEM image of SAP surface before water absorption; (**d**) SEM image of SAP surface after water absorption. (**C**) (**a**) Re-swelling ability of SAP; (**b**) water retention at different temperatures; and (**c**) swelling kinetics of SAP in distilled water. Reproduced with permission from ref. [4] © 2021 Elsevier Ltd.

Zhang et al. studied the preparation of PVPP-based superabsorbent polymer gels and their application in water retention in sandy soils. They found that using tap water as the reaction medium and acrylate and cross-linked polyvinylpyrrolidone (PVPP) (Aladdin) as raw materials, the SAPs prepared by solution polymerization achieved about 80% of their maximum water absorption within 30 min. When mixed with sandy soil at a certain temperature, the SAP effectively reduced soil water evaporation rates and improved water absorption and retention (Figure 8). The results show that mixing SAP with sandy soil can improve soil performance, enhance water retention, conserve moisture, promote plant growth, and have wide applications in desert greening projects [4].

### 4.3. Soil Domain

SAP can be used to improve acidic and saline–alkaline weakly alkaline soils, enhance the soil environment, reduce soil compaction issues, conserve moisture, and increase water absorption capacity [45]. In acidic soils, SAP can enhance the structure of microbial communities in the soil, promoting seedling emergence and growth [113]. SAP can serve as a soil conditioner, improving the physical properties of the soil, enhancing its water retention capacity, maintaining fertility, and increasing soil aeration and microbial activity, which in turn promotes plant growth and improves the soil environment [1].

Meanwhile, the application of SAP can increase soil porosity. When SAPs encounter water and swell, they reduce the drainage pore space between soil particles, effectively decreasing water infiltration from one soil layer to another, thereby enhancing water retention capacity and soil aeration [114]. Studies have found that the application of SAP can improve soil organic carbon, microbial biomass carbon, microbial biomass nitrogen content, and soil microbial activity [115]. The presence of SAP enhances the soil’s water-holding capacity and nutrient retention capabilities and also contributes to the increased activity of certain soil enzymes. Soil microbial biomass plays a crucial role in maintaining soil health, as it produces various enzymes involved in mineralization and immobilization processes, which are essential in the biogeochemical cycling of soil nutrients. Additionally, SAP may provide extra nutritional support for the growth of many cellulose-degrading soil microorganisms, promoting microbial growth in the soil and fostering microbial diversity development, thereby enhancing soil vitality and plant growth and survival rates [116].

The ability of SAP to repeatedly absorb and release water is due to its three-dimensional network structure. When combined with soil, it can alter the physical and chemical properties of the soil [113]. By enhancing water conservation ability and improving soil quality, SAP helps promote and control the release of fertilizers, thereby improving fertilizer efficiency and crop productivity, which further promotes plant growth [52]. In saline–alkaline soils, SAP has excellent water retention properties, can absorb salts from the soil, and effectively promotes seedling emergence and increases seedling survival rates in semi-arid areas, thus achieving higher yields [113]. It helps alleviate the effects of drought and optimizes irrigation by increasing water resource supply. The microplastics potentially generated during the application of SAPs can also have certain impacts on soil. MPs can affect the structure of soil ecosystems, not only compromising the overall quality of the soil environment but also posing serious threats to terrestrial biodiversity. Moreover, if they alter the microbial communities in fertile soils, they can further influence greenhouse gas emissions, leading to environmental pollution [107]. Therefore, SAPs should possess good biodegradability to prevent the excessive generation of microplastics during their application, thereby avoiding environmental contamination. Additionally, studies have found that in high-salinity soils, water-retention agents struggle to improve soil conditions and can lead to soil compaction, adversely affecting crop growth. Thus, under such environmental conditions, the salt tolerance of SAPs should be enhanced [117]. When applying SAPs, it is essential to adapt to the environment, employ site-specific strategies, and use them reasonably and efficiently to establish a favorable balance with the environment, thereby maximizing their superior performance. SAPs are being explored globally to strengthen water conservation, soil quality, and control fertilizer release, thus improving fertilizer efficiency and enhancing crop productivity.

**Figure 9:**
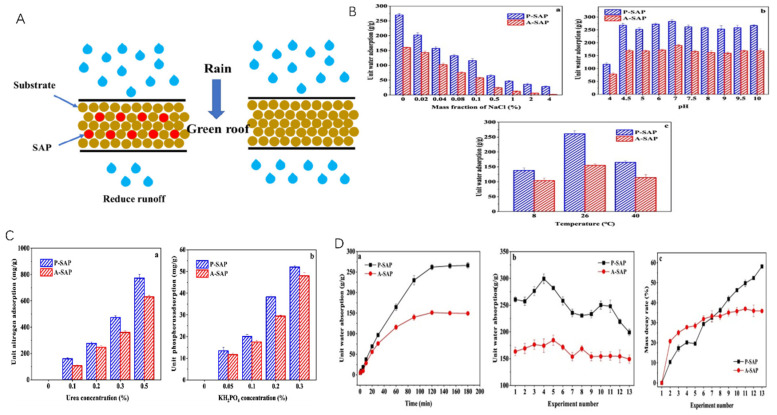
Comparison of performance between P-SAP and A-SAP: (**A**) Mechanism of action. (**B**) Comparison of the stability of the two materials in terms of (**a**) NaCl concentration, (**b**) pH, and (**c**) temperature. (**C**) Comparison of the fertilizer protection ability exhibited by the two materials: (**a**) Nitrogen fertilizer (**b**) Phosphorus fertilizer. (**D**) Comparison of (**a**) the water absorption performance, (**b**) reusability, and (**c**) mass density rate of the two materials during application. Reproduced with permission from ref. [118] © 2021 Elsevier B.V.

The use of SAP can reduce soil hardening while effectively improving the porosity of clayey soils during the water cycle process. Under drought conditions, it can alleviate the environmental impact on crops and promote their growth [45]. At the same time, the application rate of SAP also affects the soil’s resistance to erosion and its water retention capacity, effectively enhancing the soil’s water absorption ability and enabling it to release moisture under drought conditions (Figure 9) [118].

Studied showed that the water absorption and retention ability of SAP in soil and its promotion of plant growth. They found that soil containing SAP can better absorb and retain moisture while also improving soil aeration, increasing microbial activity, and enhancing the oxygen availability for plant roots, thereby promoting plant growth. The results indicated that SAP could serve as an excellent water-retaining agent in soil, fully demonstrating its water retention and absorption capabilities. Soil containing SAP has stronger water and nutrient retention abilities, improving soil conditions and promoting plant growth [111].

## 5. Conclusions and Outlook

This review systematically introduces the definition, classification, and synthesis techniques of SAP and emphasizes its important role in ecological applications, based on its excellent properties such as high water absorption and retention. The review discusses its applications in three key areas: agriculture, desertification greening, and soil, detailing the different roles SAP plays based on its structural and performance characteristics. This highlights its broad prospects in promoting the development and application of ecological fields.

The application of SAP in multiple areas brings significant benefits, primarily including economic and ecological benefits. In terms of economic benefits, research on SAP can stimulate the prosperity of industries such as chemicals and materials, create numerous job opportunities, and drive social and economic development. Ecologically, the excellent water absorption and retention properties of SAP can improve soil conditions, promote crop growth, address water scarcity in arid regions, reduce resource waste and environmental remediation costs, and encourage society to move toward a resource-friendly and environmentally sustainable direction.

SAP high water-absorbing hydrogels have promising prospects in ecological protection and resource utilization. This review explores the application of SAP in environmental issues from three aspects: material classification, preparation technologies, and applications. It summarizes the various uses of SAP in agriculture, desertification greening, and soil, including as a water-retaining agent, soil conditioner, seed additive, seed coating, and plant growth regulator fixer. These applications not only enrich the theoretical system of SAP’s ecological applications but also provide new solutions for its practical use.

Through a review of numerous references, this paper aims to comprehensively summarize and analyze the classification, preparation, and ecological applications of SAP high water-absorbing resins, providing a comprehensive perspective for research in this field. However, due to limitations in time and academic level, this review does not deeply explore the theoretical and technical details behind some key mechanisms of synthesis. In terms of technological synthesis and preparation, this review does not fully cover all viewpoints and theories in the related fields, and thus there are certain deficiencies in research depth and breadth.

## Data Availability

No new data were created or analyzed in this study.
